# Bonnesen-style Wulff isoperimetric inequality

**DOI:** 10.1186/s13660-017-1305-3

**Published:** 2017-02-14

**Authors:** Zengle Zhang, Jiazu Zhou

**Affiliations:** 1 School of Mathematics and Statistics, Southwest University, Chongqing, 400715 People’s Republic of China; 2 Southeast Guizhou Vocational College of Technology for Nationalities, Kaili, Guizhou 556000 China

**Keywords:** 52A10, 52A22, Wulff flow, Wulff isoperimetric inequality, Bonnesen-style Wulff isoperimetric inequality, reverse Bonnesen-style Wulff isoperimetric inequality

## Abstract

The Wulff isoperimetric inequality is a natural extension of the classical isoperimetric inequality (Green and Osher in Asian J. Math. 3:659-676 1999). In this paper, we establish some Bonnesen-style Wulff isoperimetric inequalities and reverse Bonnesen-style Wulff isoperimetric inequalities. Those inequalities obtained are extensions of known Bonnesen-style inequalities and reverse Bonnesen-style inequalities.

## Introduction and main results

In this paper, we restrict ourselves to considering convex bodies in the Euclidean plane $\mathbb{R}^{2}$. A set *K* is convex if, for any two points $x, y\in K$, the line segment $\overline{xy}$ joining *x* and *y* is contained in *K*, that is,

$$(1-t)x+ty\in K, \quad 0\leq t \leq1. $$

A compact convex set with non-empty interior is called a convex body. A convex body without corner points and line segments on the boundary is called an oval body.

Let $K, W$ be two convex bodies and $t\in\mathbb{R}$, the Minkowski addition $K+W$ of *K* and *W* is

$$K+W=\{x+y:x\in K, y\in W\}, $$

and the scalar multiplication *tK* of *K* is

$$t K=\{t x: x\in K\}. $$

The convex bodies *K* and *W* are homothetic if $K=x+tW$ with some $t>0$ and some $x\in\mathbb{R}^{2}$.

Being of considerable interest, for example, in studying the growth of crystals, the Wulff flow originated from the unit-speed outward normal flow. Given a planar convex domain *K* of area $A_{K}$ and perimeter $L_{K}$, by growing in the unit-speed along the direction of the outward normal, the area of the corresponding domain, which is denoted by $A_{K}(t)$, is a polynomial in *t*, which is known as the Steiner polynomial, that is,

1 $$ A_{K}(t)=A_{K}+L_{K}t+\pi t^{2}. $$

The discriminant of $A_{K}(t)=0$ is

$$\Delta(K)=L_{K}^{2}-4\pi A_{K}. $$

$\Delta(K)$ is the isoperimetric deficit of *K*, which is non-negative by the following classical isoperimetric inequality:

2 $$\begin{aligned} L_{K}^{2}-4\pi A_{K}\geq0, \end{aligned}$$

where the equality holds if and only if *K* is a disc. One can find some simplified and beautiful proofs of (2) that lead to generalizations to higher dimensions and applications to other branches of mathematics (*cf.* [1–26]).

During the 1920s, Bonnesen proved a series of inequalities of the form

3 $$ L_{K}^{2}-4\pi A_{K}\geq B_{K}, $$

where $B_{K}$ is a non-negative geometric invariant and vanishes only when *K* is a disc. The inequality of the form (3) is known as the Bonnesen-style inequality, and the typical one was proved by Bonnesen himself (*cf.* [5, 6]):

4 $$\begin{aligned} L_{K}^{2}-4\pi A_{K}\geq \pi^{2}(R-r)^{2}, \end{aligned}$$

where *R* and *r* are the radius of the minimum circumscribed disc and the radius of the maximum inscribed disc of *K*, respectively.

Many $B_{K}$s were found in the last century, and mathematicians are still working on those unknown isoperimetric deficit lower limits of geometric significance. For more details, see references [20, 24–26].

If instead *K* grows by varying the outward normal speed to be a function ${p_{W}}(\theta)$ of the direction of the unit normal, one has the Wulff flow. The area of the domain when the initial domain *K* is convex and the function ${p_{W}}(\theta)$ is a support function of the convex body *W* with area $A_{W}$ is a polynomial in *t* in this flow, called the Wulff-Steiner polynomial, that is (*cf.* [1]),

5 $$\begin{aligned} A_{K,W}(t)=A_{K}+L_{K,W} t+A_{W}t^{2}, \end{aligned}$$

where $L_{K,W}$ is the Wulff length of *∂K* with respect to *W*, and namely,

6 $$ L_{K,W} = \int_{\partial K}{p_{W}} \,ds, $$

where *∂K* is the boundary of *K*, and *s* is the arc length parameter of *∂K*. The discriminant of $A_{K,W}(t)=0$ is defined as the Wulff isoperimetric deficit (*cf.* [1]):

7 $$ \Delta_{W}(K)=L_{K,W}^{2}-4A_{K}A_{W}. $$

When domain *W* is a unit disc, the Wulff isoperimetric deficit $\Delta_{W}(K)$ is the isoperimetric deficit of *K*.

Let

$$r_{W}=\max\{t|\text{some translate of }tW\text{ is contained in }K\} $$

and

$$R_{W}=\min\{t|\text{some translate of }tW\text{ contains }K\} $$

be the W-inradius and W-outradius of *K*, respectively. It is obvious that $0< r_{W}\leq R_{W}$.

If *W* is the unit disc, then $r_{W}$ and $R_{W}$ are, respectively, the radius of the maximum inscribed disc and the radius of the minimum circumscribed disc of *K*.

We first prove the following Wulff isoperimetric inequality:

8 $$\begin{aligned} L^{2}_{K, W}-4A_{K}A_{W}\geq0, \end{aligned}$$

where the equality holds if and only if *K* and *W* are homothetic. Then we consider the inequality of the form

9 $$\begin{aligned} L^{2}_{K, W}-4A_{K}A_{W}\geq B_{W}(K), \end{aligned}$$

where $B_{W}(K)$ is an invariant of geometric significance of *K* and *W* and vanishes only when *K* and *W* are homothetic. The inequality of type (9) is called the Bonnesen-style Wulff isoperimetric inequality. Its reverse form, that is,

10 $$\begin{aligned} L^{2}_{K, W}-4A_{K}A_{W}\leq U_{W}(K), \end{aligned}$$

is called the reverse Bonnesen-style Wulff isoperimetric inequality. Here $U_{W}(K)$ is an invariant of *K* and *W*. We hope that $U_{W}(K)$ vanishes only when *K* and *W* are homothetic.

Our main results are the following.

### Theorem 1

*Let* $K, W$ *be two oval bodies in* $\mathbb{R}^{2}$ *with areas* $A_{K}$ *and* $A_{W}$, *respectively*. *Let* $r_{W}$, $R_{W}$ *be*, *respectively*, *the W*-*inradius and W*-*outradius of* *K*. *Then*

11 $$\begin{aligned} &L^{2}_{K, W}-4A_{K}A_{W} \geq A^{2}_{W}(R_{W}-r_{W})^{2}+(A_{W}R_{W}+A_{W}r_{W}- L_{K,W} )^{2}, \end{aligned}$$

12 $$\begin{aligned} &L^{2}_{K, W}-4A_{K}A_{W} \geq \frac{A_{K}A_{W}(R_{W}-r_{W})^{2}}{R_{W} r_{W}}+\frac{(R_{W} r_{W}A_{W}-A_{K})^{2}}{R_{W}r_{W}}. \end{aligned}$$

*Each equality holds if and only if* *K* *and* *W* *are homothetic*.

### Theorem 2

*Let* $K, W$ *be two oval bodies in* $\mathbb{R}^{2}$ *with areas* $A_{K}$ *and* $A_{W}$, *respectively*. *Let* $r_{W}$, $R_{W}$ *be*, *respectively*, *the W*-*inradius and W*-*outradius of* *K*. *Then*

13 $$\begin{aligned} &L^{2}_{K, W}-4A_{K}A_{W}\leq 2A_{W} L_{K,W} (R_{W}-r_{W}), \end{aligned}$$

14 $$\begin{aligned} &L^{2}_{K, W}-4A_{K}A_{W}\leq 2A_{K}L_{K,W} \biggl(\frac{1}{r_{W}}-\frac {1}{R_{W}} \biggr), \end{aligned}$$

15 $$\begin{aligned} &L^{2}_{K, W}-4A_{K}A_{W} \leq 2L_{K,W} \biggl(A_{W} R_{W}-\frac{A_{K}}{R_{W}} \biggr), \end{aligned}$$

16 $$\begin{aligned} &L^{2}_{K, W}-4A_{K}A_{W} \leq 2L_{K,W} \biggl(\frac{A_{K}}{r_{W}}-A_{W} r_{W} \biggr). \end{aligned}$$

*Each equality holds if and only if* *K* *and* *W* *are homothetic*.

## Preliminaries

The support function $p_{K}(u)$ of the convex body *K* is defined by

$$p_{K}(u)=\max\{x\cdot u|x\in K\}, $$

where $u\in S^{1}$. For simplicity, we replace $p_{K}(u)$ by $p_{K}$. For two convex bodies $K, W$, we have

17 $$\begin{aligned} p_{W}\leq p_{K} \quad\text{if and only if}\quad W \subseteq K. \end{aligned}$$

If the support functions of the convex bodies $K, W$ are denoted by $p_{K}, {p_{W}}$, respectively, and $t_{1}, t_{2}\in\mathbb{R}$, then the support function of $t_{1} K+t_{2} W$ is $t_{1} p_{K}+t_{2} {p_{W}}$.

The image of the convex body *K* at time $t\geq0$ under the normal flow having speed ${p_{W}}(u)$ (the Wulff flow associated to *W*) is $K+tW$.

Let $p_{K}$ be the support function of *K*, then

18 $$\begin{aligned} &ds=\bigl(p_{K}+p_{K}^{\prime\prime}\bigr)\,d \theta; \end{aligned}$$

19 $$\begin{aligned} &A_{K}=\frac{1}{2} \int_{S^{1}}p_{K}\bigl(p_{K}+p_{K}^{\prime\prime} \bigr)\,d\theta; \end{aligned}$$

20 $$\begin{aligned} &L_{K}= \int_{S^{1}}\bigl(p_{K}+p_{K}^{\prime\prime} \bigr)\,d\theta= \int_{S^{1}}p_{K}\,d\theta. \end{aligned}$$

### Proposition 1

Poincaré lemma [1]

*Let* *f* *be a function on* $[0,a]$ *whose first derivative is square integrable and such that*

$$f(0)=f(a)=0. $$

*Then*

21 $$\begin{aligned} \int_{0}^{a} f^{\prime}(x)^{2}\,dx\geq \biggl(\frac{\pi}{a} \biggr)^{2} \int_{0}^{a}f(x)^{2}\,dx, \end{aligned}$$

*where equality holds if and only if* $f=A\cos(\pi x/a)+B\sin(\pi x/a)$. *In particular*, *if* $a\leq\pi$, *then*

22 $$\begin{aligned} \int_{0}^{a}f^{\prime}(x)^{2}\,dx\geq \int_{0}^{a}f(x)^{2}\,dx. \end{aligned}$$

*Inequality* (22) *holds as an equality if and only if*

$$f=c\sin(\pi x/a) $$

*for some constant* *c*.

## Bonnesen-style Wulff isoperimetric inequalities

To prove our main results, we need the following lemmas.

### Lemma 1

*Let* *K*, *W* *be two convex bodies in* $\mathbb{R}^{2}$. *Let* $p_{K}(\theta)$ *and* $p_{W}(\theta)$ *be support functions of* *K* *and* *W*, *respectively*. *If* $W\subseteq K$ *such that* $p_{W}(\theta)\ne p_{K}(\theta)$, $\theta\in[\theta_{0},\theta_{0}+\pi]$ *for some* $\theta_{0}$, *then there exist* $\epsilon >0$ *and* $v\in S^{1}$ *such that* $W+\epsilon\cdot v\subset K$.

### Proof

Choose vectors $u, v\in S^{1}$ corresponding to angles $\theta, \theta_{0}+\frac{\pi}{2}$, respectively. If $\theta\in [\theta_{0}, \theta_{0}+\pi]^{c}$, the complement of $[\theta_{0}, \theta_{0}+\pi]$, since the angle between *u* and *v* is strictly greater than $\frac{\pi}{2}$, hence $u\cdot v < 0$. By (17), we have

23 $$\begin{aligned} p_{W+\epsilon\cdot v}(u)=p_{W}(u)+\epsilon(u\cdot v)< p_{K}(u) \end{aligned}$$

for $\epsilon>0$.

If $\theta\in[\theta_{0},\theta_{0}+\pi]$, since $p_{W}(\theta)\ne p_{K}(\theta)$ when $\theta\in[\theta_{0},\theta_{0}+\pi]$ and $W \subseteq K$, we have

$$p_{W}(u)< p_{K}(u). $$

For $\epsilon>0$ small enough, we have

24 $$\begin{aligned} p_{W+\epsilon\cdot v}(u)=p_{W}(u)+\epsilon(u\cdot v)< p_{K}(u). \end{aligned}$$

Hence

$$p_{W+\epsilon\cdot v}(u)< p_{K}(u), $$

for all $u\in S^{1}$, that is, $W+\epsilon\cdot v\subset K$. □

### Lemma 2

*Let* $K, W$ *be two oval bodies in* $\mathbb{R}^{2}$. *Let* $r_{W}$, $R_{W}$ *be*, *respectively*, *the W*-*inradius and W*-*outradius of* *K*. *Then the equation* $A_{K,W}(t)=0$ *has two roots* $t_{1}, t_{2}$ *such that*

25 $$\begin{aligned} t_{1}\leq-R_{W}\leq-r_{W} \leq t_{2}< 0. \end{aligned}$$

*Each inequality in* (25) *holds as an equality if and only if* *K* *and* *W* *are homothetic*. *In particular*, *when* $r_{W}\leq t\leq R_{W}$,

26 $$\begin{aligned} A_{K,W}(-t)\leq0. \end{aligned}$$

*Inequality* (26) *is strict whenever* $r_{W}< t< R_{W}$. *When* $t=r_{W}$ *or* $t=R_{W}$, *equality will occur in* (26) *if and only if* *K* *and* *W* *are homothetic*.

### Proof

There is at least one point where $\partial(r_{W}W)$ is tangent to *∂K* for $\theta\in[\theta_{0}, \theta_{0}+\pi]$ with all $\theta_{0}$. If the conclusion fails, that is, there exists $\theta_{0}$ such that $p_{r_{W}W}(\theta) \ne p_{K}(\theta) $ for $\theta\in[\theta_{0}, \theta_{0}+\pi]$, choose the vector *v* corresponding to the angle $\theta_{0}+\frac{\pi}{2}$. By Lemma 1, if we move $r_{W}W$ by *v* for $\epsilon>0$ small enough, then $r_{W}W+\epsilon\cdot v$ continues to lie in the interior of *K* and has no points of tangency. This contradicts the maximality of $r_{W}$.

By integration by parts we have

27 $$\begin{aligned} A_{K,W}(-r_{W})&=\frac{1}{2} \int_{S^{1}}(p_{K}-r_{W}{p_{W}} ) \bigl(p_{K}+p_{K}^{\prime\prime}-r_{W} \bigl({p_{W}} +p_{W} ^{\prime\prime}\bigr)\bigr)\,d\theta \\ &=\frac{1}{2} \int_{S^{1}}(p_{K}-r_{W}{p_{W}} )^{2}\,d\theta+\frac{1}{2} \int_{S^{1}}(p_{K}-r_{W}{p_{W}} ) \bigl(p_{K}^{\prime\prime}-r_{W}p_{W} ^{\prime\prime}\bigr)\,d\theta \\ &= \frac{1}{2} \int_{S^{1}}(p_{K}-r_{W}{p_{W}} )^{2}\,d\theta-\frac{1}{2} \int_{S^{1}}\bigl(p_{K}^{\prime}-r_{W}p_{W} ^{\prime}\bigr)^{2}\,d\theta. \end{aligned}$$

Let $\theta_{1}, \theta_{2},\ldots,\theta_{N}$ be points where $\partial(r_{W}W)$ are tangent to *∂K*. We can break up the right-hand side of (27) into integrals over the intervals [$\theta_{i},\theta_{i+1}$] ($1\leq i\leq N-1$). Since every set $[\theta, \theta+\pi]$ contains a point where $r_{W}W$ is tangent to *K*, we have

$$\theta_{i+1}-\theta_{i}\leq\pi. $$

Let

$$f=p_{K}-r_{W}{p_{W}}, $$

then

$$f(\theta_{i})=0 $$

at each point of tangency. Applying inequality (22) in the Poincaré lemma, we have

28 $$\begin{aligned} A_{K,W}(-r_{W})\leq0, \end{aligned}$$

where the equality holds if and only if

$$p_{K}-r_{W} {p_{W}}=c\sin\biggl( \frac{\pi\theta}{\alpha}\biggr). $$

Since the convex body *K* contains *tW*, then

$$c\sin\biggl(\frac{\pi\theta}{\alpha}\biggr)=p_{K}-r_{W}{p_{W}} \geq0 $$

for all *θ*. This leads to

$$c=0, $$

that is, *K* and *W* are homothetic. In a similar way, we have

29 $$\begin{aligned} A_{K,W}(-R_{W})\leq0, \end{aligned}$$

where the equality holds if and only if *K* and *W* are homothetic. Thus the equation $A_{K,W}(t)=0$ has two roots $t_{1}, t_{2}$, and

$$t_{1}+t_{2}=-\frac{ L_{K,W} }{A_{W}}< 0, \qquad t_{1}t_{2}= \frac{A_{K}}{A_{W}}>0, $$

and therefore

$$t_{1}< 0,\qquad t_{2}< 0. $$

Therefore

$$t_{1}\leq-R_{W}\leq-r_{W}\leq t_{2}. $$

In particular, according to (5), when $r_{W}\leq t\leq R_{W}$, we have

$$A_{K,W}(-t)\leq0. $$

If $r_{W} < t< R_{W}$, then $t_{1}<-t<t_{2}$. Inequality (26) is strict. Therefore, equality occurs in (26) only when $t=r_{W}$ or $t=R_{W}$, that is, *K* and *W* are homothetic. Lemma 2 is proved. □

### Remark

Inequality (26) has been mentioned in Green and Osher’s work (cf. [1]) without proof. For general convex bodies, Luo, Xu and Zhou [17] have also obtained inequality (26) by the integral geometry method. However, it is difficult to obtain the equality condition of inequality (26) for general convex bodies. Via the method of convex geometric analysis, a complete proof of inequality (26) with equality condition is given in [9].

By (28) or (29), the sufficient condition for root existence of equation $A_{K, W}(t)=0$ is that the discriminant of $A_{K, W}(t)=0$ is non-negative. We obtain the following Wulff isoperimetric inequality.

### Corollary 1

*Let* $K, W$ *be two oval bodies in* $\mathbb{R}^{2}$ *with areas* $A_{K}, A_{W}$, *then*

$$L_{K,W}^{2}-4A_{K}A_{W}\geq0, $$

*the equality holds if and only if* *K* *and* *W* *are homothetic*.

### Proof of Theorem 1

By inequalities (28), (29), we have, respectively,

30 $$\begin{aligned} &A_{K}- L_{K,W} r_{W}+A_{W}r_{W}^{2} \leq0 , \end{aligned}$$

31 $$\begin{aligned} &A_{K}- L_{K,W} R_{W}+A_{W}R_{W}^{2} \leq0 . \end{aligned}$$

Then inequalities (30), (31) can be, respectively, rewritten as

$$\begin{aligned} &{-}2A_{K}A_{W}\geq2A_{W}^{2}r_{W}^{2}-2 L_{K,W} r_{W}A_{W}, \\ &{-}2A_{K}A_{W}\geq2A_{W}^{2}R_{W}^{2}-2 L_{K,W} R_{W}A_{W}. \end{aligned}$$

Therefore, we have

$$\begin{aligned} L_{K,W} ^{2}-4A_{K}A_{W}\geq{}& L_{K,W} ^{2}+2A_{W}^{2} r_{W}^{2}+2A_{W}^{2}R_{W}^{2}-2 L_{K,W} r_{W}A_{W}-2 L_{K,W} R_{W}A_{W} \\ ={}&A_{W}^{2}r_{W}^{2}+A_{W}^{2}R_{W}^{2}-2A_{W}^{2}r_{W}R_{W}+ L_{K,W} ^{2}+A_{W}^{2}r_{W}^{2}+A_{W}^{2}R_{W}^{2} \\ &{}+2A_{W}^{2}r_{W}R_{W}-2 L_{K,W} r_{W}A_{W}-2 L_{K,W} R_{W}A_{W} \\ ={}&A_{W}^{2}(R_{W}-r_{W})^{2}+(A_{W}R_{W}+A_{W}r_{W}- L_{K,W} )^{2}, \end{aligned}$$

where the equality holds if and only if the equalities of (28), (29) hold, that is, *K* and *W* are homothetic. This proves inequality (11).

Inequalities (30), (31) can also be rewritten, respectively, as follows:

$$\begin{aligned} &L_{K,W} r_{W}\geq A_{W}r_{W}^{2}+A_{K}, \\ &L_{K,W} R_{W}\geq A_{W}R_{W}^{2}+A_{K}. \end{aligned}$$

Therefore

$$\begin{aligned} L_{K,W} ^{2}r_{W}R_{W}-4A_{K}A_{W}r_{W}R_{W} \geq{}& A_{W}^{2}r_{W}^{2}R_{W}^{2}+A_{K}A_{W}r_{W}^{2}+A_{K}A_{W}R_{W}^{2}+A_{K}^{2} \\ &{}-4A_{K}A_{W}r_{W}R_{W} \\ ={}&A_{K}A_{W}(R_{W}-r_{W})^{2}+(R_{W}r_{W}A_{W}-A_{K})^{2}. \end{aligned}$$

Hence, we have

$$L_{K,W} ^{2}-4A_{K}A_{W}\geq \frac{A_{K}A_{W}(R_{W}-r_{W})^{2}}{R_{W}r_{W}}+\frac{(R_{W}r_{W}A_{W}-A_{K})^{2}}{R_{W}r_{W}}, $$

where the equality holds if and only if *K* and *W* are homothetic. Inequality (12) is proved. □

Let *W* be the unit disc, then $L_{K,W}^{2}=L_{K}^{2}$, $A_{W}=\pi$. Therefore we have the following.

### Corollary 2

*Let* *K* *be an oval body in* $\mathbb{R}^{2}$ *with area* $A_{K}$ *and perimeter* $L_{K}$. *Let* *r* *and* *R* *be*, *respectively*, *the radius of the maximum inscribed disc and the radius of the minimum circumscribed disc of* *K*. *Then*

32 $$\begin{aligned} &L_{K}^{2}-4\pi A_{K}\geq \pi^{2}(R-r)^{2}+( \pi R+\pi r- L_{K} )^{2}, \end{aligned}$$

33 $$\begin{aligned} &L_{K}^{2}-4\pi A_{K}\geq \frac{\pi A_{K}(R-r)^{2}}{Rr}+ \frac{(\pi Rr-A_{K})^{2}}{Rr}. \end{aligned}$$

*Each equality holds if and only if* *K* *is a disc*.

It should be noted that (32) is obtained in [24], which is stronger than the Bonnesen isoperimetric inequality (4).

## Reverse Bonnesen-style Wulff isoperimetric inequalities

To prove reverse Bonnesen-style Wulff isoperimetric inequalities in Theorem 2, we need the following Wulff isoperimetric inequalities.

### Lemma 3

*Let* $K, W$ *be two oval bodies in* $\mathbb{R}^{2}$ *with areas* $A_{K}$ *and* $A_{W}$. *Let* $r_{W}$, $R_{W}$ *be*, *respectively*, *the W*-*inradius and W*-*outradius of* *K*. *Then*

34 $$\begin{aligned} &r_{W}\leq\frac{2A_{K}}{ L_{K,W} } \leq\sqrt{ \frac{A_{K}}{A_{W}}}\leq \frac{ L_{K,W} }{2A_{W}}\leq R_{W}, \end{aligned}$$

35 $$\begin{aligned} &\frac{A_{K}}{A_{W}R_{W}}\leq\frac{2A_{K}}{ L_{K,W} } \leq \sqrt{ \frac{A_{K}}{A_{W}}} \leq\frac{ L_{K,W} }{2A_{W}} \leq \frac{A_{K}}{A_{W}r_{W}}. \end{aligned}$$

*Each equality holds if and only if* *K* *and* *W* *are homothetic*.

### Proof

The Wulff isoperimetric inequality

$$L_{K,W} ^{2}-4A_{K}A_{W}\geq0 $$

can be rewritten as

36 $$ \frac{2A_{K}}{ L_{K,W} } \leq\sqrt{\frac{A_{K}}{A_{W}}}\leq \frac{ L_{K,W} }{2A_{W}}. $$

Each inequality holds as an equality if and only if *K* and *W* are homothetic. Recalling (6), (18) and (19), we have

$$\begin{aligned} &2A_{K}= \int_{S^{1}}p_{K}\bigl(p_{K}+p_{K}^{\prime\prime} \bigr)\,d\theta, \\ &r_{W} L_{K,W} = \int_{S^{1}}r_{W}{p_{W}} \bigl(p_{K}+p_{K}^{\prime\prime}\bigr)\,d\theta. \end{aligned}$$

By the definition of $r_{W}$, we have

$$r_{W} {p_{W}} \leq p_{K} $$

for all *θ*, which leads to

$$r_{W} L_{K,W}\leq2A_{K}, $$

that is,

37 $$ r_{W}\leq\frac{2A_{K}}{ L_{K,W} }, $$

where the equality holds if and only if $r_{W}{p_{W}}=p_{K}$ for all *θ*, that is, *K* and *W* are homothetic. By the definition of $L_{K,W}$ in (6), we have

$$L_{K,W} = \int_{S^{1}}{p_{W}} \bigl(p_{K}+p_{K}^{\prime\prime} \bigr)\,d\theta = \int_{S^{1}}p_{K}\bigl({p_{W}} +p_{W} ^{\prime\prime}\bigr)\,d\theta. $$

Via the area formula (19), we have

$$2A_{W}R_{W}= \int_{S^{1}}R_{W}{p_{W}} \bigl({p_{W}} +{p_{W}} ^{\prime\prime}\bigr)\,d\theta \geq \int_{S^{1}}p_{K} \bigl({p_{W}} +{p_{W}} ^{\prime\prime}\bigr)\,d\theta=L_{K,W}. $$

Hence, we have

38 $$ \frac{ L_{K,W} }{2A_{W}}\leq R_{W}, $$

where the equality holds if and only if $R_{W}{p_{W}}=p_{K}$ for all *θ*, that is, *K* and *W* are homothetic.

By (36), (37) and (38), we have

$$r_{W}\leq\frac{2A_{K}}{ L_{K,W} } \leq \sqrt{\frac{A_{K}}{A_{W}}}\leq \frac{L_{K,W}}{2A_{W}}\leq R_{W}. $$

Inequalities (34) are proved.

Inequalities (38), (37) can, respectively, be rewritten as

$$\frac{A_{K}}{A_{W}R_{W}}\leq\frac{2A_{K}}{ L_{K,W} }, \qquad\frac{ L_{K,W} }{2A_{W}}\leq \frac{A_{K}}{A_{W}r_{W}}. $$

Together with (36) and the above inequalities, inequalities (35) follow. □

### Proof of Theorem 2

By inequalities (34), we have

39 $$\begin{aligned} 2A_{W}L_{K,W} r_{W} \leq4A_{K}A_{W}\leq L_{K,W}^{2} \leq2A_{W}L_{K,W} R_{W}, \end{aligned}$$

then

$$L_{K,W} ^{2}-4A_{K}A_{W} \leq2A_{W} L_{K,W} R_{W}-2A_{W} L_{K,W} r_{W}=2A_{W} L_{K,W} (R_{W}-r_{W}), $$

where the equality holds if and only if each equality of (34) holds, that is, *K* and *W* are homothetic. This is inequality (13).

By inequalities (35), we have

40 $$\begin{aligned} 2\frac{A_{K}L_{K,W}}{R_{W}}\leq4A_{K}A_{W}\leq L_{K,W} ^{2} \leq 2\frac{A_{K}L_{K,W}}{r_{W}}, \end{aligned}$$

then

$$L_{K,W} ^{2}-4A_{K}A_{W}\leq \frac{2A_{K} L_{K,W} }{r_{W}}-\frac{2A_{K} L_{K,W} }{R_{W}} =2A_{K}L_{K,W} \biggl( \frac{1}{r_{W}}-\frac{1}{R_{W}} \biggr), $$

where the equality holds if and only if each equality of (35) holds, then *K* and *W* are homothetic. This is inequality (14).

By (39) and (40), we obtain

$$\begin{aligned} 2\frac{A_{K}L_{K,W}}{R_{W}}\leq4A_{K}A_{W}\leq L_{K,W}^{2} \leq2A_{W}L_{K,W} R_{W}, \end{aligned}$$

then

$$L_{K,W} ^{2}-4A_{K}A_{W} \leq2A_{W} L_{K,W} R_{W}-\frac{2A_{K} L_{K,W}}{R_{W}}. $$

According to the equality conditions of (39) and (40), the equality holds for (15) if and only if *K* and *W* are homothetic. This gives inequality (15).

By (39) and (40) again, we get

41 $$\begin{aligned} 2A_{W}L_{K,W} r_{W}\leq4A_{K}A_{W} \leq L_{K,W}^{2} \leq 2\frac{A_{K}L_{K,W}}{r_{W}}, \end{aligned}$$

then

$$\begin{aligned} L_{K,W} ^{2}-4A_{K}A_{W}\leq \frac{2A_{K} L_{K,W} }{r_{W}}-2A_{W} L_{K,W} r_{W}. \end{aligned}$$

From the equality conditions of (39) and (40) again, the equality of (16) holds if and only if *K* and *W* are homothetic. This gives inequality (16). Theorem 2 is proved. □

Let *W* be a unit disc. Direct consequences of Theorem 2 are as follows.

### Corollary 3

*Let* *K* *be an oval body in* $\mathbb{R}^{2}$ *with area* $A_{K}$ *and perimeter* $L_{K}$. *Let* *r* *and* *R* *be*, *respectively*, *the radius of the maximum inscribed disc and the radius of the minimum circumscribed disc of* *K*. *Then*

42 $$\begin{aligned} &L_{K}^{2}-4\pi A_{K}\leq 2\pi L_{K} (R-r), \end{aligned}$$

43 $$\begin{aligned} &L_{K}^{2}-4\pi A_{K}\leq 2A_{K} L_{K} \biggl(\frac{1}{r}-\frac{1}{R} \biggr), \end{aligned}$$

44 $$\begin{aligned} &L_{K}^{2}-4\pi A_{K}\leq 2L_{K} \biggl(\pi R-\frac{A_{K}}{R} \biggr), \end{aligned}$$

45 $$\begin{aligned} &L_{K}^{2}-4\pi A_{K}\leq 2L_{K} \biggl(\frac{A_{K} }{r}-\pi r \biggr). \end{aligned}$$

*Each equality holds if and only if* *K* *is a disc*.

The reverse Bonnesen-style inequality (42) is obtained by Bokowski, Heil, Zhou, Ma and Xu (*cf.* [4, 27]).
